# Cytokine profiles in adults with imported malaria

**DOI:** 10.1038/s41598-023-36212-2

**Published:** 2023-06-26

**Authors:** Charles de Roquetaillade, Cédric Laouenan, Jean-Paul Mira, Carine Roy, Marie Thuong, Élie Azoulay, Didier Gruson, Frédéric Jacobs, Juliette Chommeloux, François Raffi, Laurent Hocqueloux, Patrick Imbert, Vincent Jeantils, Jean-Luc Delassus, Sophie Matheron, Catherine Fitting, Jean-François Timsit, Fabrice Bruneel

**Affiliations:** 1 Department of Anesthesiology and Critical Care, Hôpital Lariboisière, FHU PROMICE, DMU Parabol, AP-HP Nord, Paris, France; 2 INSERM UMR 942 MASCOT, Université de Paris-Cité, Paris, France; 3 Département Epidémiologie Biostatistiques et Recherche Clinique, AP-HP, INSERM, Centre d’Investigation Clinique-Epidémiologie Clinique 1425, Hôpital Bichat, Paris, France; 4 UMR 1137, Université de Paris-Cité, INSERM, IAME, Hôpital Bichat, AP-HP, Paris, France; 5 Service de medecine intensive-reanimation, Hôpital Cochin, AP-HP Centre, Paris, France; 6 Université Paris Cité, Paris, France; 7 Institut Cochin, INSERM U1016, CNRS UMR 8104, Université Paris Cité, AP-HP, Paris, France; 8 Service de medecine intensive-reanimation, Centre Hospitalier René Dubos, Pontoise, France; 9 Service de medecine intensive-reanimation, Hôpital Saint-Louis, Hôpitaux Universitaires Paris-Nord, AP-HP, Paris, France; 10 Service de medecine intensive-reanimation, Hôpital Pellegrin-Tripode, Bordeaux, France; 11 Centre de Recherche Cardio-Thoracique, CHU Bordeaux, Bordeaux, France; 12 Service de medecine intensive-reanimation, Hôpital Antoine Béclère, Université Paris-Saclay, AP-HP, Clamart, France; 13 Service de medecine intensive reanimation, Institut de Cardiologie, ICAN, Groupe Hospitalier Pitié-Salpêtrière, Sorbonne Université, AP-HP, Paris, France; 14 CHU de Nantes, CIC UIC 1413 Inserm, Nantes, France; 15 Service de maladies infectieuses, Centre Hospitalier Régional d’Orléans, Orléans, France; 16 Centre de vaccinations internationales, Hôpital d’instruction des armees Bégin, Saint-Mandé, France; 17 Service de maladies infectieuses, Hôpital Jean Verdier, AP-HP, Bondy, France; 18 Service de medecine interne et de maladies infectieuses, Centre hospitalier intercommunal Robert-Ballanger, Aulnay-sous-Bois, France; 19 Service de maladies infectieuses et tropicales, Hôpital Bichat, GHU Paris Nord, AP-HP, Paris, France; 20 Unité cytokines et inflammation, Institut Pasteur, Paris, France; 21 Service de medecine intensive et reanimation (MI2), Hôpital Bichat, Paris, France; 22 IAME, Université de Paris, INSERM U1137, AP-HP, Paris, France; 23 Service de reanimation, Centre Hospitalier de Versailles, Hôpital André Mignot, Le Chesnay, France

**Keywords:** Biomarkers, Cytokines, Parasitology

## Abstract

The increase in worldwide travel is making imported malaria a growing health concern in non-endemic countries. Most data on the pathophysiology of malaria come from endemic areas. Little is known about cytokine profiles during imported malaria. This study aimed at deciphering the relationship between cytokine host response and malaria severity among imported cases in France. This study reports cytokine profiles in adults with *Plasmodium falciparum* malaria included in the PALUREA prospective study conducted between 2006 and 2010. The patients were classified as having uncomplicated malaria (UM) or severe malaria (SM), with this last further categorized as very severe malaria (VSM) or less severe malaria (LSM). At hospital admission, eight blood cytokines were assayed in duplicate using Luminex^®^ technology: interleukin (IL)-1α, IL-1β, IL-2, IL-4, IL-10, tumor necrosis factor (TNF)α, interferon (IFN)γ, and macrophage migration inhibitory factor (MIF). These assays were repeated on days 1 and 2 in the SM group. Of the 278 patients, 134 had UM and 144 SM. At hospital admission, over half the patients had undetectable levels of IL-1α, IL-1β, IL-2, IL-4, IFNγ, and TNFα, while IL-10 and MIF were significantly higher in the SM vs. the UM group. Higher IL-10 was significantly associated with higher parasitemia (R = 0.32 [0.16–0.46]; *P* = 0.0001). In the SM group, IL-10 elevation persisting from admission to day 2 was significantly associated with subsequent nosocomial infection. Of eight tested cytokines, only MIF and IL-10 were associated with disease severity in adults with imported *P. falciparum* malaria. At admission, many patients had undetectable cytokine levels, suggesting that circulating cytokine assays may not be helpful as part of the routine evaluation of adults with imported malaria. Persisting high IL-10 concentration was associated with subsequent nosocomial infection, suggesting its possible interest in immune monitoring of most severe patients.

## Introduction

*Plasmodium falciparum* malaria has been a scourge for humanity since antiquity and remains so today. Between 2001 and 2015, important worldwide mobilization contributed to a 30% reduction of its global incidence and a decrease in related mortality by 47%^1^. However, it caused 619 000 deaths in 2021 with a stable incidence over the last few years according to the World Health Organization (WHO) most recent report^2^. Imported malaria, defined as contracted in an endemic area but developing in a non-endemic country, is a growing health concern in the northern hemisphere due to the increase in worldwide travel. Among European countries, France has the most cases, about 5000 annually, of which 10–15% are severe and the great majority are due to *P. falciparum*. In France, severe imported malaria has ranged a fatality rate of 3–11%^1,3,4^. Given the available medical resources, lower rates should be achievable^1^.

Taken together, previous studies have suggested a role of pro-inflammatory cytokines magnitude and imbalance in pro-inflammatory and anti-inflammatory cytokines as determinants of falciparum malaria severity and mortality^5–9^. However, patients with imported falciparum malaria differ from those in endemic areas. Most are adults with no past *P. falciparum* exposure^4^. Importantly, despite better baseline health and greater access to healthcare resources, severe forms of malaria are more common. These differences may be related to host factors that affect the immune response. We know little about the immune response to imported malaria. The few available studies on cytokine signature during imported malaria were done in small and heterogeneous populations^10,11^.

*Plasmodium falciparum* infection triggers strong inflammatory and immune responses similar to those seen in bacterial infections^6,10,12^ despite the many differences in underlying pathophysiology. Endothelial sequestration of parasitized erythrocytes is unique to falciparum malaria and may cause the main manifestations of severe forms. The relative roles for inflammation vs. immune responses in promoting erythrocyte sequestration remain debated.

This study reported the cytokine results of PALUREA study, and aimed at deciphering the relationship between cytokine host response and malaria severity among imported cases in France.

## Patients and methods

### Study design and patients

The present study is a pre-specified ancillary analysis of data from the previously reported prospective multicenter PALUREA cohort study conducted in France between November 2006 and March 2010 and designed to investigate several host- and parasite-related biomarkers, with the objective of improving the evaluation of severity^13^. The study protocol was approved for all participating centers on May 2, 2006, by the ethics committee of the Saint-Louis University Hospital, Paris, France (CCPPRB, approval #2006/24). PALUREA complied with French regulations, the Declaration of Helsinki, and Good Clinical Practices. Written informed consent was obtained before inclusion from each patient, or next of kin if the patient had lost competency; according to French law, incapacitated patients with no available next of kin were included then asked, as soon as they regained competency, whether they consented to stay in the study.

As described in detail elsewhere^13^, the participating centers enrolled consecutive patients with either uncomplicated malaria (UM) or severe malaria (SM). We defined SM as *P. falciparum* malaria requiring admission to the intensive care unit (ICU) and fulfilling the modified 2000 WHO criteria for severe malaria in adults^14,15^ at admission or within the first 2 ICU days (see Supplementary Table S1). In the SM group, based on French guidelines^16^, we predefined two subgroups, namely, very severe malaria (VSM) and less severe malaria (LSM). VSM was defined as any of the following: coma, shock, acidosis, hyperlactatemia > 5 mmol/L, or respiratory distress, within the first 72 h after ICU admission. LSM was defined as SM with none of the criteria for VSM. UM was defined by no requirement for ICU admission and absence of criteria for SM. Isolated jaundice or isolated parasitemia > 4% was considered as UM. Patients were managed according to international guidelines at the time of recruitment and all patients were given intravenous quinine as antimalarial treatment, since artesunate was not recommended at that time.

### Cytokine assays

We assayed a panel of pro- and anti-inflammatory cytokines in blood samples collected in 5-mL heparin tubes, at hospital admission (day 0) in all patients and on days 1 and 2 in patients with SM. Tubes were transferred to the Cochin University Hospital (Paris, France) within 2 h, in cooled bags, then centrifuged for 10 min at 3500 rpm at 4 °C to allow plasma collection. The following eight cytokines were assayed in duplicate using Luminex technology (Bio-Plex 200 Systems, Waltham, MA): interleukin (IL)-1α, IL-1 β, IL-2, IL-4, IL-10, tumor necrosis factor (TNF)-α, interferon-γ (IFNγ), and macrophage migration inhibitory factor (MIF). Those cytokines were chosen by a panel of experts (FB, JFT, JPM) given their known role in malaria pathogenesis. In patients with SM, the same cytokines were assayed using the same method on days 1 and 2.

#### Statistics

Continuous data are described as median and interquartile range [IQR], and qualitative variables as number and percentage. Cytokine values were also described with boxplots according to the different patient groups.

For cytokines below the detection limit (DL), imputation was made using the single value of DL/2. This method is commonly used as an imputation method in the presence of detection limit and has been reported in similar studies^17^.

Comparisons between two groups (UM vs SM or LSM vs VSM) were performed with Student t-test or Mann–Whitney test as appropriate for continuous variables, and with chi-square test or Fisher exact test as appropriate for categorical variables. To compare plasmatic cytokines level between the three groups UM, LSM and VSM, the non-parametric Kruskal–Wallis’s test was used.

The association between plasmatic cytokines level and some of biological data was measured using Spearman correlation coefficients.

All statistical analyses were performed using SAS version 9.4 software (SAS Institute Inc., Cary, NC).

## Results

### Clinical characteristics (Table 1)

**Table 1:** Baseline characteristics at admission and hospital mortality in the groups with uncomplicated malaria (UM) and severe malaria (SM).

	Total (n = 278)	UM (n = 134)	SM (n = 144)	*P*	LSM (n = 76)	VSM (n = 68)	*P*
Age, years, median [IQR]	42 [32–52]	38 [29–47]	45 [35–57]	< 0.01	43 [29–56]	48 [38–57]	0.03
Male, n (%)	193 (69.4)	97 (72.4)	96 (66.7)	0.30	46 (60.5)	50 (73.5)	0.10
Black African n (%)	148 (53.2)	79(59.0)	69 (47.9)	0.08	50 (65.8)	19 (27.9)	< 0.01
Lives in an endemic area, n (%)	45 (16.4)	25 (18.9)	20 (14.1)	0.28	11 (14.5)	9 (13.6)	0.88
African country source, n (%)	266 (96.7)	130 (97.7)	136 (95.8)	0.50	74 (97.4)	62 (93.9)	0.41
Antimalarial chemoprophylaxis, n (%)	81 (29.3)	40 (29.9)	41 (28.9)	0.86	22 (28.9)	19 (28.8)	0.98
Immune suppression, n (%)	23 (8.4)	7 (5.3)	16 (11.3)	0.07	6 (8.1)	10 (14.7)	0.21
At least one comorbidity, n (%)	31 (11.3)	8 (6.0)	23 (16.3)	< 0.01	9 (12.3)	14 (20.6)	0.18
Time from symptom onset to hospital admission, days, median [IQR]	4 [2–6]	3 [2–6]	4 [3–6.5]	< 0.01	4.5 [3.0–6.0]	4.0 [2.5–7.0]	0.80
Characteristics at admission							
Number of WHO criteria on day 1, median [IQR]	1 [0–2]	0 [0–0]	2 [1–3]	–	1 [1–2]	3 [2–5]	< 0.01
Parasitemia on day 1, %, median [IQR]	2.5 [0.3–9.0]	0.5 [0.1–2.1]	8.0 [3.8–15.0]	< 0.01	8.0 [4.0–14.0]	9.5 [3.3–20.0]	0.18
*pf*HRP2 on day 1 (ng/mL), median, [IQR]	263 [40–983]	60.7 [12–261]	834.4 [304–2099]	< 0.01	693 [193–1422]	1110 [475–3402]	0.01
Outcome							
Bacterial coinfection < 48 h, n (%)	11 (4.0)	1 (0.7)	10 (6.9)	< 0.01	2 (2.6)	8 (11.8)	0.04
Nosocomial infection, n (%)	23 (8.3)	2 (1.5)	21 (14.6)	< 0.01	5 (6.6)	16 (23.5)	< 0.01
Death, n (%)	8 (5.2)	0	8 (5.2)	NA			

*SM* severe malaria, *UM* uncomplicated malaria, *LSM* less severe malaria, *VSM* very severe malaria, *IL* interleukin, *MIF* macrophage migration inibitory factor, *SAPS II* Simplified Acute Physiology Score version II, *SOFA* Sequential Organ Failure Assessment, *WHO* World Health Organization, *NA* not applicable.

#### All patients, and groups with uncomplicated malaria versus severe malaria

Of the 278 patients with cytokine data, 134 had UM and 144 SM, including 68 with VSM and 76 with LSM (see the Supplementary Fig. S1 for Flow-Chart). The SM group was characterized by older age, a higher proportion of patients with comorbidities, a longer time from symptom onset to hospital admission, higher parasitemia on day 0, and a higher *pf*HRP2 level (Table 1). Coinfection at admission was present in 10 SM patients vs. a single UM patient. The rate of adherence to antimalarial chemoprophylaxis was not different between the two groups (*p* = 0.86). Nosocomial infections were significantly more common in the SM group (*P* < 0.01). All 8 patients who died had SM (8/144, 5.5%).

#### Patients with severe malaria and sub-groups with very severe malaria versus less severe malaria

VSM patients were older and less likely to be black than LSM patients (Table 1). Time from symptom onset to hospital admission was not different between the two sub-groups (*P* = 0.8), and use of antimalarial chemoprophylaxis was reported in near 29% in both sub-groups (*P* = 0.98). The higher *pf*HRP2 value in the VSM sub-group despite no significant parasitemia difference with the LSM sub-group on day 0 suggested a greater sequestered parasite biomass^18^.

#### Circulating cytokine levels at hospital admission (day 0) (Fig. 1 and Table 2)

**Figure 1:**
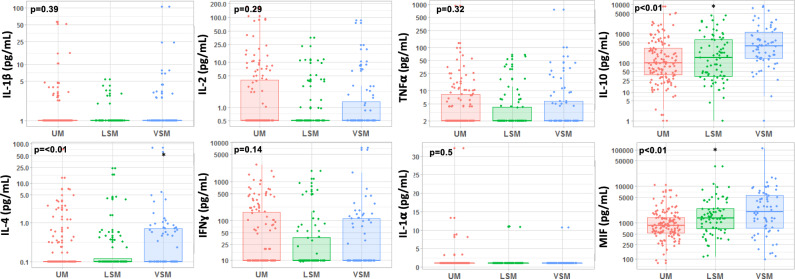
Boxplot of circulating cytokine levels at admission (D0) in patients with uncomplicated malaria (UM), less severe malaria (LSM), and very severe malaria (VSM). All cytokines are expressed in pg/mL. Kruskal–Wallis test for intergroup distribution, with *P* values < 0.05 considered significant.

**Table 2:** Cytokine profiles in patients with severe malaria according to severity.

n (%) or median [IQR]	Total (n = 278)	UM (n = 134)	SM (n = 144)	*P* value	LSM (n = 76)	VSM (n = 68)	*P* value
IL-1b detectable	30 (10.8)	14 (10.4)	16 (11.1)	0.858	6 ( 7.9)	10 (14.7)	0.19
IL-1 b, pg/mL	1.00 [1.00–1.00]	1.00 [1.00–1.00]	1.00 [1.00–1.00]	0.879	1.00 [1.00–1.00]	1.00 [1.00–1.00]	0.17
IL-1a detectable	9 (3.2)	6 (4.5)	3 (2.1)	0.321	2 ( 2.6)	1 (1.5)	1.0
IL-1 a, pg/mL	1.00 [1.00–1.00]	1.00 [1.00–1.00]	1.00 [1.00–1.00]	0.268	1.00 [1.00–1.00]	1.00 [1.00–1.00]	0.62
IL-2 detectable	82 (29.6)	43 (32.1)	39 (27.3)	0.380	18 (24.0)	21 (30.9)	0.36
IL-2, pg/mL	0.50 [0.50–1.90]	0.50 [0.50–4.18]	0.50 [0.50–0.97]	0.163	0.50 [0.50–0.50]	0.50 [0.50–1.42]	0.43
IL-4 detectable	78 (28.1)	30 (22.4)	48 (33.3)	0.042	19 (25.0)	29 (42.6)	0.02
IL-4, pg/mL	0.10 [0.10–0.31]	0.10 [0.10–0.10]	0.10 [0.10–0.50]	0.038	0.10 [0.10–0.17]	0.10 [0.10–0.70]	0.02
IL-10 detectable	275 (98.9)	133 (99.3)	142 (98.6)	1.0	75 (98.7)	67 (98.5)	1.00
IL-10, pg/mL	159.76 [45.93–570.42]	100.79 [39.16–320.65]	223.63 [52.21–769.52]	0.001	149.48 [34.77–629.11]	375.62 [142.35–1176.2]	< 0.01
TNF a detectable, n (%)	95 (34.2)	50 (37.3)	45 (31.3)	0.286	21 (27.6)	24 (35.3)	0.32
TNF a, pg/mL	2.00 [2.00–6.23]	2.00 [2.00–8.33]	2.00 [2.00–5.22]	0.277	2.00 [2.00–4.26]	2.00 [2.00–5.76]	0.29
IFNg detectable	105 (37.8)	53 (39.6)	52 (36.1)	0.554	25 (32.9)	27 (39.7)	0.40
IFNg, pg/mL	9.50 [9.50–97.80]	9.50 [9.50–171.28]	9.50 [9.50–73.61]	0.179	9.50 [9.50–38.06]	9.50 [9.50–116.66]	0.12
MIF detectable	278 (100)	134 (100)	144 (100)	NA	76 (100)	68 (100)	NA
MIF, pg/mL	1207.3 [567.45–2296.9]	842.10 [533.15–1410.4]	1511.0 [681.28–4330.7]	< 0.01	1357.4 [681.71–2461.2]	2002.2 [681.28–5968.4]	0.14

*UM* uncomplicated malaria, *SM* severe malaria, *LSM* less severe malaria, *VSM* very severe malaria, *IL* interleukin, *TNF* tumor necrosis factor, *IFN* interferon, *MIF* macrophage migration inhibitory factor, *NA* not applicable.

Levels were often undetectable for six cytokines (IL-1α, IL-1 β, IL-2, IL-4, IFNγ, and TNFα) (Table 2, Fig. 1). IL-4 was more often detectable in the SM group than in the UM group (*P* = 0.04); for other cytokines, the proportions of patients with detectable levels were not different across groups and sub-groups (Table 2). IL-4, IL-10, and MIF were significantly higher in patients with greater disease severity (Fig. 1). Only IL-10 was significantly associated with parasitemia (R = 0.32 [0.16–0.46]; *P* = 0.0001). IL-4, IFNγ, TNFα, IL-10, and MIF were significantly but only weakly associated with the *pf*HRP2 level at admission (Supplementary Table S2). None of the cytokine levels were associated with albuminemia or hemoglobin level at hospital admission. Higher IL-10 and MIF were significantly associated with higher procalcitonin (data not shown). The cytokine profile at admission did not differ between the patients who died (n = 8) and those who survived (Supplementary Table S3).

#### Changes in circulating IL-10 and MIF levels during the first 3 ICU days (Fig. 2)

**Figure 2:**
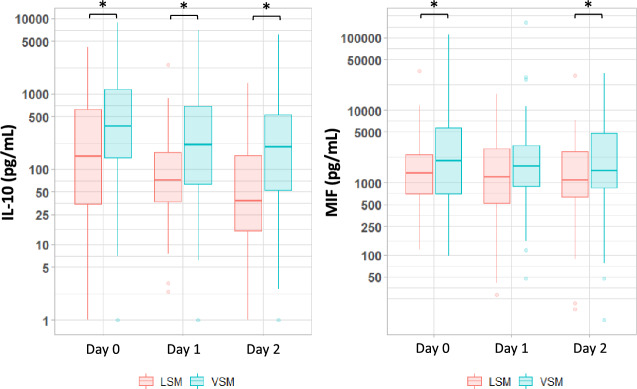
Boxplot of circulating cytokine levels in the sub-groups with less severe malaria (LSM) and very severe malaria (VSM) during the first 3 days following ICU admission. All cytokines are expressed in pg/mL. Sub-group comparison using the t-test between groups, with *P* values < 0.05 considered significant. Note that no significant difference was observed within subgroups over time for IL10 nor MIF.

In the 144 patients with SM, the eight cytokines were assayed on the first ICU day then on the next 2 days. Median IL-10 levels were significantly higher in the VSM vs. the LSM sub-group on days 1 and 2 (day 1: 209.36 [58.44–693.21] vs. 72.49 [35.01–169.52], *P* < 0.01; day 2: 195.20 [52.34–532.60] vs. 37.78 [15.20–151.35] on day 2, *P* < 0.01; Fig. 2). Plasma IL-10 concentration did not vary across time within each subgroup. The median MIF values were significantly higher in the VSM sub-group vs. the LSM sub-group on day 2 but not on day 1 (day 1: 1676.0 ng/mL [898.16–3321.60] vs. 1206.90 ng/mL [507.98–2960.50], *P* = 0.12; day 2: 1462.20 ng/mL [825.94–4898.10] vs. 1083.20 ng/mL [631.91–2687.50], *P* = 0.02). MIF concentration was stable over time within each subgroup. Levels of the other cytokines did not differ significantly between the VSM and LSM sub-groups (data not shown). Interestingly, high IL-10 levels persisting over the 3 days were more common in patients with versus without nosocomial infections (Supplementary Table S4).

## Discussion

In this preplanned analysis of data from the prospective PALUREA observational cohort study^13^, we evaluated circulating levels of eight cytokines in 278 adults admitted for imported malaria, including 134 with UM and 144 with SM. At hospital admission, IL-1α, IL-1 β, IL-2, IL-4, IFNγ, and TNFα were undetectable in over half the patients, whereas IL-10 and MIF levels were detectable and significantly higher in the SM group. Higher IL-10 levels were significantly associated with higher parasitemia.

Cytokines play a pivotal role in the host response to *P. falciparum* infection. We found no meaningful associations with disease severity and frequently undetectable levels for six of the eight tested cytokines (IL-1α , IL-1 β, IL2, IL4, TNFα, and IFNγ). A technical issue as the reason for the often-undetectable cytokine levels is unlikely, given the duplicate assays at a central, highly experienced laboratory. The most reasonable interpretation is that levels of the tested cytokines were low in most patients at hospital admission.

A good balance between the pro-inflammatory and anti-inflammatory host responses is crucial to parasite eradication without further organ damage due to runaway inflammation. IL-10 is a fundamental anti-inflammatory cytokine that plays an important regulatory function in malarial models^8,19^. In our study IL-10 was associated with malaria severity as in several studies^9,11,20–22^. The exact role of IL-10 in malaria pathogenesis is debated. In one experimental study, IL-10 demonstrated its ability to inhibit endothelial ICAM-1 expression, with a resulting decrease in parasite sequestration^23^. Conversely, IL-10 also exerts potent anti-inflammatory effects, which may paradoxically promote parasite growth^24^. The direction of the association between IL-10 and parasite burden cannot be determined from our data: IL-10 elevation may have been triggered by a heavy parasite burden, and/or, parasite replication may have been promoted by high IL-10 levels. Finally, the strong immunosuppressive effect of IL-10 on circulating monocytes^25,26^ may lead to increased susceptibility to bacterial infections^27^. In our group with SM, patients with high IL-10 levels during the first 3 ICU days had a significantly greater number of nosocomial infections.

MIF, one of the first cytokines identified, is constitutively expressed by a broad spectrum of cells and tissues. MIF contributes to regulate immune responses and is an important mediator of inflammatory diseases in mammals^28^. Recently, the discovery of a *P. falciparum*-encoded MIF ortholog (PfMIF) drew attention to the role for MIF in the pathophysiology of malaria^29,30^. MIF exerts pro-inflammatory properties that may increase the cytoadherence of parasitized erythrocytes, resulting in greater disease severity. MIF also inhibits erythroid, multipotential, and granulocyte–macrophage progenitor-derived colony formation (CFU-GM) and may therefore be involved in the pathophysiology of malarial anemia^31^. We found that higher MIF levels were associated with greater disease severity, in keeping with findings in Zambian children^21^ and Indian infants and adults^32^. In contrast to the work from Zambia, our study showed no association between MIF levels and anemia. However, the absence of PfMIF assays in our study precludes conclusions about the role for MIF in malarial anemia. To our knowledge, no studies have investigated the levels of both MIF isoforms (human MIF and PfMIF) and their relationship with malaria severity. Therapeutic interventions designed to neutralize PfMIF have shown promise for protecting against malaria^33,34^.

### Perspectives

The main benefits expected from assessing immune responses in critically ill patients with malaria are the characterization of specific immune dysfunctions and the identification of patients at high risk for nosocomial infections. Higher IL-10 levels may be associated with worse immune dysfunction and greater susceptibility to nosocomial infections. In preclinical studies, high IL-10 levels were associated with decreases in the release of other cytokines and in class II major histocompatibility complex expression on monocytes^35^. In our population, persistently high IL-10 levels were associated with subsequent nosocomial infections, as previously reported in sepsis but not in malaria^26^. Detailed monitoring of both cytokines and membrane receptors associated with immune suppression such as mHLA-DR is a promising approach and deserves further investigations^27^.

### Limitations

Our study has several limitations. First, the small number of patients who died may have limited our ability to detect statistically significant differences in cytokine profiles between non-survivors and survivors. However, other markers of disease severity such as WHO severity criteria showed limited differences between groups. Secondly, it is possible that the timing of cytokines sampling may have limited our ability to detect differences between groups. Indeed, patients had to be sampled on admission to the ICU, but the time between ICU admission and cytokine blood sampling may have varied among patients. . However, our study was a pragmatic study with the aim of identifying stratification biomarkers that could be used in routine practice. Finally, the participating centers were expert centers in the management of malaria, which may limit further generalization of our results.

## Conclusion

Among the eight cytokines tested in this large cohort of adults with imported falciparum malaria, only MIF and IL-10 were higher in patients with greater disease severity. Our findings suggest that cytokine assays at ICU admission may have a limited role for the diagnosis and assessment of severity. Whether monitoring the potent anti-inflammatory cytokine IL-10 helps to identify patients at high risk for developing nosocomial infection deserves further investigation.

## Supplementary Information

Supplementary Information.

## Data Availability

The datasets used and/or analyzed during the current study available from the corresponding author on reasonable request.

## References

[1] Bouchaud O, Bruneel F, Caumes E, Houzé S, Imbert P, Pradines B (2020). Management and prevention of imported malaria. 2018 update of the 2007 French clinical guidelines. Méd. Mal. Infect..

[2] World malaria report 2022 [Internet]. [cited 2023 Feb 10]. Available from: https://www.who.int/teams/global-malaria-programme/reports/world-malaria-report-2022

[3] El Ket N, Kendjo E, Thellier M, Assoumou L, Potard V, Taieb A (2020). Propensity score analysis of artesunate versus quinine for severe imported *Plasmodium falciparum* Malaria in France. Clin. Infect. Dis. Off. Publ. Infect. Dis. Soc. Am..

[4] Bruneel F, Tubach F, Corne P, Megarbane B, Mira JP, Peytel E (2010). Severe imported falciparum Malaria: A cohort study in 400 critically ill adults. PLoS ONE.

[5] Severe Malaria. *Trop. Med. Int. Health ***19**(s1), 7–131 (2014). 10.1111/tmi.12313_2 25214480

[6] Grau GE, Piguet PF, Vassalli P, Lambert PH (1989). Tumor-necrosis factor and other cytokines in cerebral malaria: experimental and clinical data. Immunol. Rev..

[7] Mandala WL, Msefula CL, Gondwe EN, Drayson MT, Molyneux ME, MacLennan CA (2017). Cytokine profiles in malawian children presenting with uncomplicated malaria, severe malarial anemia, and cerebral malaria. Clin. Vaccine Immunol. CVI..

[8] Kumar R, Ng S, Engwerda C (2019). The role of IL-10 in malaria: A double edged sword. Front. Immunol..

[9] Day NP, Hien TT, Schollaardt T, Loc PP, Chuong LV, Chau TT (1999). The prognostic and pathophysiologic role of pro- and antiinflammatory cytokines in severe malaria. J. Infect. Dis..

[10] Kern P, Hemmer CJ, Van Damme J, Gruss HJ, Dietrich M (1989). Elevated tumor necrosis factor alpha and interleukin-6 serum levels as markers for complicated *Plasmodium falciparum* malaria. Am. J. Med..

[11] Wroczyńska A, Nahorski W, Bakowska A, Pietkiewicz H (2005). Cytokines and clinical manifestations of malaria in adults with severe and uncomplicated disease. Int. Marit. Health.

[12] Clark IA, Cowden WB (2003). The pathophysiology of falciparum malaria. Pharmacol. Ther..

[13] Bruneel F, Tubach F, Mira JP, Houze S, Gibot S, Huisse MG (2016). Imported falciparum malaria in adults: host- and parasite-related factors associated with severity. The French prospective multicenter PALUREA cohort study. Intensive Care Med..

[14] Organization WH. Severe falciparum malaria. *Trans. R Soc. Trop. Med. Hyg*. **94**(Supplement_1), 1–90 (2000).

[15] Dondorp, A., Nosten, F., Stepniewska, K., Day, N., & White, N. South East Asian Quinine Artesunate Malaria Trial (SEAQUAMAT) group. Artesunate versus quinine for treatment of severe falciparum malaria: a randomised trial. *Lancet Lond Engl.* **366**(9487), 717–25 (2005). 10.1016/S0140-6736(05)67176-0 16125588

[16] Société de Pathologie Infectieuse de Langue Française, Collège des Universitaires de Maladies Infectieuses et Tropicales, Société Française de Médecine des Armées, Société Française de Parasitologie, Société Française de Pédiatrie, Société de Médecine des Voyages, et al. [Management and prevention of imported Plasmodium falciparum malaria (Revision 2007 of the 1999 Consensus Conference). Long text in French]. *Med. Mal. Infect*. **38**(2), 68–117 (2008). 10.1016/j.medmal.2007.11.009 18646361

[17] Lubin JH, Colt JS, Camann D, Davis S, Cerhan JR, Severson RK (2004). Epidemiologic evaluation of measurement data in the presence of detection limits. Environ. Health Perspect..

[18] Dondorp AM, Desakorn V, Pongtavornpinyo W, Sahassananda D, Silamut K, Chotivanich K (2005). Estimation of the total parasite biomass in acute falciparum malaria from plasma PfHRP2. PLOS Med..

[19] Freitas do Rosario AP, Langhorne J (2012). T cell-derived IL-10 and its impact on the regulation of host responses during malaria. Int. J. Parasitol..

[20] Lyke KE, Burges R, Cissoko Y, Sangare L, Dao M, Diarra I (2004). Serum levels of the proinflammatory cytokines interleukin-1 beta (IL-1beta), IL-6, IL-8, IL-10, tumor necrosis factor alpha, and IL-12(p70) in Malian children with severe Plasmodium falciparum malaria and matched uncomplicated malaria or healthy controls. Infect. Immun..

[21] Thuma PE, van Dijk J, Bucala R, Debebe Z, Nekhai S, Kuddo T (2011). Distinct clinical and immunologic profiles in severe malarial anemia and cerebral malaria in Zambia. J. Infect. Dis..

[22] Sobota RS, Goron AR, Berry AA, Bailey JA, Coulibaly D, Adams M (2022). Serologic and cytokine profiles of children with concurrent cerebral malaria and severe malarial anemia are distinct from other subtypes of severe malaria. Am. J. Trop. Med. Hyg..

[23] Kossodo S, Monso C, Juillard P, Velu T, Goldman M, Grau GE (1997). Interleukin-10 modulates susceptibility in experimental cerebral malaria. Immunology.

[24] Weidanz WP, Batchelder JM, Flaherty P, LaFleur G, Wong C, van der Heyde HC (2005). Plasmodium chabaudi adami: use of the B-cell-deficient mouse to define possible mechanisms modulating parasitemia of chronic malaria. Exp. Parasitol..

[25] Fumeaux T, Pugin J (2002). Role of interleukin-10 in the Intracellular sequestration of human leukocyte antigen-DR in monocytes during septic shock. Am. J. Respir. Crit. Care Med..

[26] Monneret G, Finck ME, Venet F, Debard AL, Bohé J, Bienvenu J (2004). The anti-inflammatory response dominates after septic shock: association of low monocyte HLA-DR expression and high interleukin-10 concentration. Immunol. Lett..

[27] de Roquetaillade C, Dupuis C, Faivre V, Lukaszewicz AC, Brumpt C, Payen D (2022). Monitoring of circulating monocyte HLA-DR expression in a large cohort of intensive care patients: Relation with secondary infections. Ann. Intensive Care.

[28] Calandra T, Roger T (2003). Macrophage migration inhibitory factor: A regulator of innate immunity. Nat. Rev. Immunol..

[29] Sun T, Holowka T, Song Y, Zierow S, Leng L, Chen Y (2012). A Plasmodium-encoded cytokine suppresses T-cell immunity during malaria. Proc. Natl. Acad. Sci. U S A..

[30] Cordery DV, Kishore U, Kyes S, Shafi MJ, Watkins KR, Williams TN (2007). Characterization of a *Plasmodium falciparum* macrophage-migration inhibitory factor homologue. J. Infect. Dis..

[31] Martiney JA, Sherry B, Metz CN, Espinoza M, Ferrer AS, Calandra T (2000). Macrophage migration inhibitory factor release by macrophages after ingestion of *Plasmodium chabaudi*-infected erythrocytes: Possible role in the pathogenesis of malarial anemia. Infect. Immun..

[32] Jain V, McClintock S, Nagpal AC, Dash AP, Stiles JK, Udhayakumar V (2009). Macrophage migration inhibitory factor is associated with mortality in cerebral malaria patients in India. BMC Res Notes..

[33] Baeza Garcia A, Siu E, Sun T, Exler V, Brito L, Hekele A (2018). Neutralization of the *Plasmodium*-encoded MIF ortholog confers protective immunity against malaria infection. Nat. Commun..

[34] Baeza Garcia A, Siu E, Du X, Leng L, Franke-Fayard B, Janse CJ (2021). Suppression of *Plasmodium* MIF-CD74 signaling protects against severe malaria. FASEB J..

[35] de Waal MR, Abrams J, Bennett B, Figdor CG, de Vries JE (1991). Interleukin 10(IL-10) inhibits cytokine synthesis by human monocytes: An autoregulatory role of IL-10 produced by monocytes. J. Exp. Med..

