# Supplementary material for: Cytokine profiles in adults with imported malaria
Source: Sci Rep. 2023 Jun 26;13:10347. doi: 10.1038/s41598-023-36212-2 (PMC10293253; doi:10.1038/s41598-023-36212-2)
Supplement: Supplementary file 1 — Supplementary Information. [file 41598_2023_36212_MOESM1_ESM.docx]

**Cytokine Signature in Adults with Imported Malaria**

Charles de ROQUETAILLADE (1), Cedric LAOUENAN (2), Jean-Paul MIRA (3), Carine ROY (2), Marie THUONG (4), Elie AZOULAY (5), Didier GRUSON (6), Frederic JACOBS (7), Charles-Edouard LUYT (8), Francois RAFFI (9), Laurent HOCQUELOUX (10), Patrick IMBERT (11), Vincent JEANTILS (12), Jean-Louis DELASSUS (13), Sophie MATHERON (14), Catherine FITTING (15), Jean-François TIMSIT (16), Fabrice BRUNEEL (17)

**Supplementary Material**

**PALUREA Study Group: list of the collaborators (including authors)**

**Hopital Bichat Claude Bernard**, Paris: M. Wolff, B. Mourvillier, C. Aubron, L. Bouadma and JF. Timsit (ICU), S. Matheron, E. d’Ortenzio, R. Matra, E. Bouvet, J. Tourret, J. Dorchies, S. Males, C. Rioux, R. Flicoteaux and P. Yeni (Infectious Diseases Unit), C. Choquet, F. Spingler, E. Kargougou and E. Casalino (Emergency Unit), S. Houze and J. Le Bras (Parasitology and Malaria National Reference Center), MG. Huisse and V. Ollivier (Hematology), A. Kalloumeh and B. Grandchamp (Biochemistry), F. Tubach, Y. Miri and C. Roy (Département d’Epidémiologie et Recherche Clinique)

**Hopital d’Angers**: E. Fresnoy, F. Grelon, P. Asfar, A. Kouatchet and A. Mercat (ICU), ML. Joly-Guillou, M. Kempf and M. Eveillard (Microbiology)

**Hopital Paul Morel, Vesoul**: P. Daoudal (ICU), A. Royer (Parasitology)

**Hopital d’Orleans**: I. Runge, A. Mathonnet and T. Boulain (ICU), L. Bret (Parasitology), L. Hocqueloux, JM. Segalin, C. Mille, M. Niang and T. Prazuck (Infectious Diseases Unit)

**Hopital Henri Mondor**, **Creteil**: M. Fortin, F. Schortgen and C. Brun Buisson (ICU), F. Botterel and S. Bretagne (Parasitology)

**Hopital de Tourcoing**: O. Leroy, H. Georges, A. Meybeck and B. Guery (ICU), P. Patoz and C. Dhennain (Parasitology)

**Hopital Charles Nicolle**, **Rouen**: D. Chakarian, K. Azougagh and G. Bonmarchand (ICU), P. Abboud, L. Favennec and JF. Lemeland (Parasitology)

**Hopital Laveran, Marseille**: E. Peytel, R. Petrognani, A. Nau, A. Puidupin and JP. Carpentier (ICU), F. Simon (Infectious Diseases Unit), E. Garnotel and JL. Moalic (Parasitology)

**Hopital Jean Verdier, Bondy**: L. Tual, R. Amathieu and G. Dhonneur (ICU), M. N’Diaye and V. Jeantils (Infectious Diseases Unit), C. Chassaignon, AL. Guillerm, D. Riche, N. Javaud, F. Pevirieri, J. Benkel, D. Luis, MC. Bon, G. Lenoir and B. Bernot (Emergency Unit), A. Collignon, D. Bemba and I. Poilane (Parasitology)

**Hopital de la Pitie Salpetriere, Paris**: JL. Trouillet, CE. Luyt, M. Mirabel and J. Chastre (ICU), P. Hochedez, A. Perignon and E. Caumes (Infectious Diseases Unit), M. Thellier, and M. Danis (Parasitology), A. Faussart and J. Nafziger (Biology)

**Hopital de Limoges**: H. Gastinne and N. Pichon (ICU), D. Ajzenberg and ML. Darde (Parasitology)

**Hopital Antoine Beclere, Clamart**: F. Jacobs and F. Brivet (ICU), A. Veyradier and A. Marfaing-Koka (Parasitology and Hematology)

**Hopital de Grenoble**: JF. Timsit and L. Hammer (ICU), D. Maubon and H. Pelloux (Parasitology)

**Hopital Cochin**, **Paris**: F. Pene, J. Charpentier, S. Marque, C. Rousseau and JP. Mira (ICU)

**Hopital André Mignot**, **Versailles**: F. Bruneel, G. Troche (ICU), O. Eloy and C. Palette (Parasitology and Biology), A. Therby, S. Monnier and A. Greder-Bellan (Infectious Diseases Unit)

**Hopital de Rangueil, Toulouse**: P. Cougot (ICU)

**Hopital Emile Muller, Mulhouse**: K. Kuteifan, Y. Mootien and P. Guiot (ICU), JM. Delarbre and MH. Kiefer (Microbiology and Parasitology)

**Hopital Ambroise Paré, Boulogne**: B. Page and A. Vieillard Baron (ICU), J. Dunand (Parasitology)

**Hopital de Saint Germain en Laye**: Y. Loubieres, JL. Ricome (ICU), JY. Peltier and Y. Giudicelli (Parasitology and Biology)

**Hopital Begin, Saint Mandé**: C. Pelletier and JM. Rousseau (ICU), P. Imbert, C. Rapp, F. Mechai, R. Barruet and T. Debord (Infectious Diseases Unit), JE. Pilo and JD. Cavallo (Biology)

**Hopital Purpan, Toulouse**: O. Angles and M. Genestal (ICU)

**Hopital Hotel Dieu, Nantes**: C. Bretonnière, L. Nicolet, L. Jalin and D. Villers (ICU), JP. Talarmin, O. Grossi and F. Raffi (Infectious Diseases Unit), F. Gay-Andrieu and T. Bompoil (Parasitology)

**Hopital Pontchaillou, Rennes**: C. Camus, A. Gros and Y. Le Tulzo (ICU), M. Revest, F. Fily, JM. Chapplain, P. Tattevin and C. Michelet (Infectious Diseases Unit), S. Chevrier and C. Guigen (Parasitology)

**Hopital Lariboisiere, Paris**: B. Megarbane and F. Baud (ICU), JM. Launay, C. Gourmel and F. Derouin (Biology and Parasitology)

**Hopital Saint Louis, Paris**: S. de Miranda, G. Thiery, S. Legriel, E. Azoulay and B. Schlemmer (ICU), N. Colin de Verdieres, M. Lagrange-Xelot, S. Gallien, N. De Castro, J. Pavie, M. Lafaurie and JM. Molina (Infectious Diseases Unit), C. Sarfati and F. Derouin (Parasitology)

**Hopital Gui de Chauliac, Montpellier**: P. Corne and P. Jonquet (ICU), JF Schved, C. Biron and C. Bret (Hematology)

**Hopital Lapeyronie, Montpellier**: K. Klouche and P. Beraud (ICU), JP. Cristol (Biology)

**Hopital Pellegrin, Bordeaux**: D. Gruson, B. Herpe, M. Delacre and Y. Castaing (ICU), P. Fialon and P. Vincendeau (Parasitology)

**Hopital Avicenne, Bobigny**: F. Vincent, P. Karoubi and Y. Cohen (ICU), H. Cordel, V. Prendki, J. Cailhol, S. Abgrall, H. Gros, C. Fantinato and O. Bouchaud (Infectious Diseases Unit), F. Cymbalista, Y Senghor and R. Durand (Parasitology)

**Hopital Delafontaine, Saint Denis**: N. Memain, M. Thuong, C. Adrie, L. Darques, G. Moret and F. Fraisse (ICU), N. Godineau and C. Chaplain (Parasitology), M. Prevel and RJ. Mary (Emergency Unit)

**Hopital Victor Dupouy, Argenteuil**: G. Plantefeve, G. Bleichner and H. Mentec (ICU), F. Leturdu (Biology and Parasitology)

**Hopital de l’Archet, Nice**: H. Hyvernat and G. Bernardin (ICU), P. Delaunay and P. Marty (Parasitology)

**Hopital Saint Antoine, Paris**: JL. Baudel, E. Maury and G. Offenstadt (ICU), JL. Meynard J. Pacanowski, Z. Ouazene, D. Bollens, MC. Meyohas, L. Fonquerine and PM. Girard (Infectious Diseases Unit), G. Belkadi, D. Magne and P. Roux (Parasitology)

**Hopital Louis Mourier, Colombes**: L. Rusel, JD. Ricard and D. Dreyfuss (ICU), E. Mortier and P. Vinceneux (Infectious Diseases Unit), M. Bloch and G. Galeazzi (Parasitology).

**Hopital de la Côte de Nacre, Caen**: P. Charbonneau (ICU), C. Duhamel and R. Leclercq (Parasitology)

**Hopital d’Aulnay sous-Bois**: JL. Delassus and D. Malbec (Infectious Diseases Unit), D. Lusina and H. Broutier (Parasitology)

**Hopital Necker, Paris**: C. Charlier, F. Lanternier, H. Coignard and O. Lortholary (Infectious Diseases Unit), ME. Bougnoux (Parasitology)

**Centre Médical de l’Institut Pasteur de Paris**: PH. Consigny and A. Simons de Fanti (Infectious Diseases Unit), AS. Leguern (Parasitology)

**Hôpital de Nancy**: S. Gibot (ICU) and F. Massin (Immunology)

**Supplementary Table 1.** Criteria for severe malaria according to the modified 2000 World Health Organization definition

| **Clinical criteria** |
| --- |
| **Impaired consciousness**: Glasgow Coma Scale score <11 and/or **≥ two convulsions** |
| **Respiratory distress**: requirement for noninvasive and/or endotracheal mechanical ventilation or spontaneous breathing with PaO_2_ <60 mm Hg (if FiO_2_ ≥0.21)**†**, and/or respiratory rate >32/min |
| **Circulatory collapse**: systolic blood pressure <80 mm Hg despite adequate volume repletion |
| **Abnormal bleeding** |
| **Jaundice**: clinical jaundice or bilirubin >50 μmol/L |
| **Macroscopic hemoglobinuria**: if unequivocally related to acute malaria (patients with blackwater fever are not included) |
| **Laboratory criteria** |
| **Severe anemia**: hemoglobin <5 g/dL |
| **Hypoglycemia**: blood glucose <2.2 mmol/L |
| **Acidemia** (pH <7.35) or **acidosis** (serum bicarbonate <15 mmol/L) |
| **Hyperlactatemia**: arterial lactate >5 mmol/L |
| **Hyperparasitemia** ≥4% |
| **Renal impairment**: serum creatinine >265 µmol/L or blood urea nitrogen >17 mmol/L |

**Supplementary Table 2.** Correlation between cytokine levels and parasite burden on admission (day 0) in the whole population

| **Day 0 parasitemia** | **R^2^** | **p** | **Day 0 *pf*HRP2** | **R^2^** | **p** |
| --- | --- | --- | --- | --- | --- |
| **IL1**α | -0.12 [-0.28 ; 0.04] | 0.14 | **IL1**α | -0.05 [-0.22 ; 0.13] | 0.61 |
| **IL1**β | 0.02 [-0.14 ; 0.19] | 0.79 | **IL1**β | 0.17 [-0.00 ; 0.34] | 0.05 |
| **IL-2** | -0.05 [-0.22 ; 0.11] | 0.52 | **IL-2** | 0.07 [-0.11 ; 0.24] | 0.45 |
| **IL-4** | 0.10 [-0.06 ; 0.26] | 0.22 | **IL-4** | 0.18 [0.01 ; 0.34] | 0.04 |
| **IL-10** | 0.32 [0.16 ; 0.46] | <0.01 | **IL-10** | 0.39 [0.22 ; 0.52] | < 0.01 |
| **MIF** | 0.05 [-0.12 ; 0.21] | 0.57 | **MIF** | 0.19 [0.02 ; 0.36] | 0.03 |
| **IFN**γ | 0.10 [-0.07 ; 0.26] | 0.26 | **IFN**γ | 0.27 [0.10 ; 0.42] | <0.01 |
| **TNF**α | 0.02 [-0.15 ; 0.18] | 0.85 | **TNF**α | 0.22 [0.05 ; 0.38] | 0.01 |

**Supplementary Table 3.** Immunologic response on admission according to survival outcome in the whole population

| **Cytokine** | **Survivors (n=270)** | **Non survivors (n=8)** | **p** |
| --- | --- | --- | --- |
| **IL1**α | 1.00 [1.0-1.0] | 1.00 [1.0-1.0] | 0.60 |
| **IL1**β | 1.00 [1.0-1.0] | 1.00 [1.0-1.0] | 0.32 |
| **IL-2** | 0.50 [0.5-2.2] | 0.50 [0.5-0.5] | 0.07 |
| **IL-4** | 0.10 [0.1-0.3] | 0.10 [0.1-0.7] | 0.57 |
| **IFN**γ | 9.50 [9.5-103.7] | 9.50 [9.5-9.5] | 0.15 |
| **TNF**α | 2.00 [2.0-6.2] | 2.00 [2.0-6.7] | 0.75 |
| **IL-10** | 147.55 [45.0-570.4] | 371.7 [205.0-620.6] | 0.10 |
| **MIF** | 1207.3 [567.5-2287.1] | 3564.9 [721.4-8784.6] | 0.27 |

**Supplementary Table 4.** Evolution of circulating IL10 level during the first 3 days after ICU admission between severe patients presenting subsequent nosocomial infection and others

| Cytokines | Nosocomial infection (n=23) | No nosocomial infection (n=121) | P |
| --- | --- | --- | --- |
| IL10 day 0 | 278.5 [63.1 - 579.8] | 222.3 [52.2 - 816.9] | 0.62 |
| IL10 day 1 | 191.7 [111.7 - 471.0] | 110.3 [40.0 - 301.6] | 0.10 |
| IL10 day 2 | 406.3 [136.0 - 544.6] | 68.2 [17.6 - 266.2] | <0.01 |

**Supplementary Figure 1.** **Flow-chart**

**
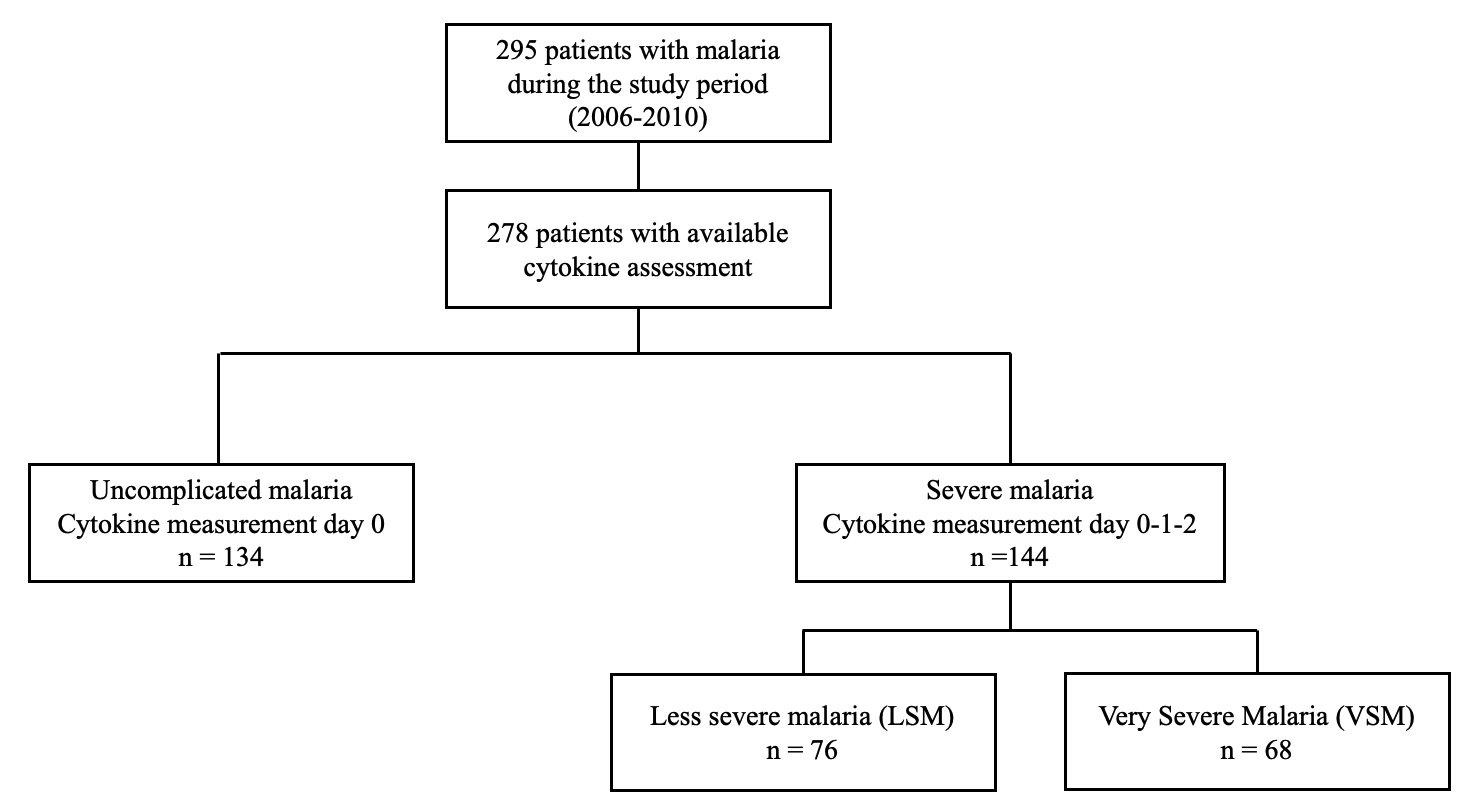
**
